# Standardized approach to median arcuate ligament syndrome and laparoscopic release: A case series

**DOI:** 10.1016/j.sipas.2022.100115

**Published:** 2022-07-28

**Authors:** Aneetinder Mann, Tyler McKechnie, Margherita Cadeddu, Jacques Tittley

**Affiliations:** a Division of Vascular Surgery, Department of Surgery, McMaster University, Hamilton, Ontario, Canada; b Division of General Surgery, Department of Surgery, McMaster University, Hamilton, Ontario, Canada

**Keywords:** Median arcuate ligament syndrome, Laparoscopy, General surgery, Vascular surgery

## Abstract

**Background:**

Median arcuate ligament syndrome (MALS) is a rare disorder characterized by compression of the celiac axis. Recently, laparoscopic approaches to median arcuate ligament release (MALR) have been described. The purpose of this study is to evaluate our institutional experience and outcomes with laparoscopic MALR.

**Methods:**

Retrospective analysis of all patients who underwent laparoscopic MALR between January 2018 and April 2019 at a single institution. A 14-question postoperative survey was administered via telephone. Peri-operative morbidity and mortality, as well as operative times were assessed.

**Results:**

Nine patients underwent laparoscopic MALR. Five patients were discharged on post-operative day one. Average length of stay was 2.1 days (+/-1.96). There were no reported intra-operative complications. Average procedure time was 92.4 min (+/-31.4), which decreased over time (p=0.046).

**Conclusions:**

This study describes a standardized approach to laparoscopic MALR that is safe and provides early relief of symptoms.

## Introduction

Median arcuate ligament syndrome (MALS) is a rare disorder characterized by chronic and recurrent abdominal pain caused by compression of the celiac axis and celiac plexus [1]. The true incidence of median arcuate ligament syndrome is unclear, however studies have demonstrated the incidence of celiac artery compression on computed tomography angiography to be approximately 7.3% in asymptomatic patients [2,3].

The median arcuate ligament is a fibrous bridge that extends from the diaphragmatic crura arising at L1-L4 and traverses the aorta anteriorly. The celiac axis typically branches between T11 and L1, however variations in its origin or a more inferiorly positioned median arcuate ligament may predispose to celiac axis compression [1]. The celiac plexus is located adjacent to the median arcuate ligament and thus symptomology arising from celiac axis compression may be secondary to both ischemic and neuropathic mechanisms [4], [5], [6], [7], [8].

Patients with celiac artery compression often present with postprandial epigastric abdominal pain, and a number of other symptoms including food fear, postprandial fullness, nausea, vomiting, and weight loss. Given the symptomology, diagnosis can be difficult and is often one of exclusion following a number of investigations including endoscopy and imaging studies. Advanced vascular imaging (computed tomography or magnetic resonance angiography) may demonstrate celiac artery compression [1]. Moreover, dynamic vascular studies including arteriography or duplex ultrasound with inspiratory and expiratory phases may be performed [1]. Additional procedures including celiac ganglion nerve block may be performed to both confirm the diagnosis and provide symptom relief. A good response to celiac ganglion nerve block can be predictive of success following surgical decompression with ganglionectomy [7].

Classically surgical decompression was performed via midline laparotomy that inherently carries significant morbidity. In the largest case series to date, open decompression alone resulted in symptom relief for 56% of their study population [8,9]. More recently, laparoscopic release has demonstrated greater relief of symptoms, shorter recovery, decreased postoperative pain and postoperative morbidity (e.g., ileus, blood loss, bowel obstruction) when compared to its open counterpart [10]. Moreover, small single institution studies have demonstrated successful short-term and long-term outcomes with laparoscopic median arcuate ligament release (MALR), with symptom relief occurring in 70-90% of cases [11,12]. Furthermore, studies have emphasized the importance of celiac plexus ganglionectomy during MALR as a subset of patients have reported complete resolution of symptoms despite evidence of ongoing residual celiac artery stenosis or occlusion on post-operative vascular imaging [6].

The purpose of this study is to describe our institutional approach to laparoscopic median arcuate ligament release in terms of preoperative assessment, operative technique, postoperative outcomes and patient reported outcomes.

## Material and methods

### Study design and inclusion criteria

A single institution retrospective case series was performed at St. Joseph's Healthcare Hospital in Hamilton, Ontario, Canada for all patients who underwent laparoscopic MALR between January 2018 and April 2019. All patients were between the ages of 18 and 80, and able to provide written and informed consent. Patients who did not participate in the follow-up telephone survey were excluded.

### Preoperative assessment

Preoperatively, all patients are assessed by a single surgical team consisting of a general surgeon and vascular surgeon. Preoperative investigations included esophagoduodenoscopy followed by computed tomography angiography to assess for classic features of median arcuate ligament syndrome including celiac artery compression with a characteristic hooked appearance and post-stenotic dilatation [1]. Moreover, the majority of patients underwent celiac plexus block by interventional radiology to assess for symptom resolution prior to advancement for surgery. If patient's experienced symptom relief with the celiac plexus block, they were considered for laparoscopic MALR.

### Surgical technique

**Fig. 1:**
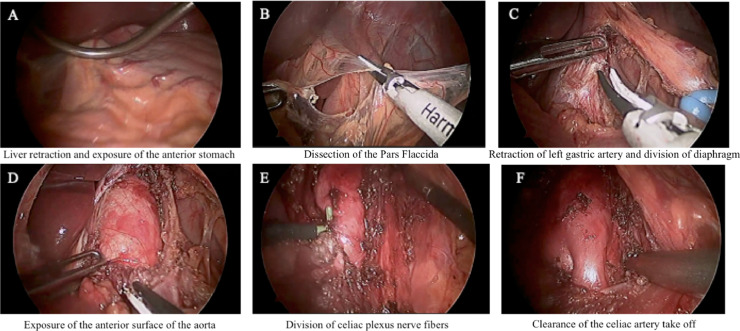
Intraoperative photos from a laparoscopic median arcuate ligament release.

The patient is placed supine in split-leg positioning with arms tucked bilaterally. Laparoscopy is undertaken in the usual fashion and capnopneumoperitoneum is established via Veress needle entry at Palmar's point. A laparoscope is introduced under direct visualization in the supraumbilical area. Additional 5mm ports are placed, two in the left upper quadrant and one in the right upper quadrant 10cm below the costal margin. A liver retractor is introduced to retract the left lobe of the liver. Dissection is started by dividing Pars Flaccida, the left gastric artery is then identified and isolated using a vessel loop, which is subsequently used for retraction. An area of diaphragm above the expected origin of the celiac trunk is identified, and its fibres are divided transversely using an energy device. The anterior aspect of the aorta is cleared superior to the celiac trunk. Using a combination of hook cautery and bipolar energy, the diaphragmatic muscle fibres are then cleared laterally to the suspected course of the celiac trunk to the level of the left gastric artery. The celiac artery is identified, and fibres are cleared along the anterior aspect of the trunk for a distance of 3cm. Particular attention is made to ensure division of all bands of the celiac ganglion and median arcuate ligament anteriorly (Fig. 1).

### Postoperative care

Patients are admitted to the inpatient surgical ward postoperatively and are assessed daily in terms of symptoms including pain, nausea and food tolerance. Once tolerating an adequate diet and pain control is achieved, patients are discharged home and seen in follow-up four-to-six weeks postoperatively to further assess symptomology.

### Telephone survey

Patients were consented to participate in a telephone survey consisting of 14 questions assessing current symptomology, changes or improvements in symptoms following surgery, and patient satisfaction (Appendix 1). The current survey was developed by the researchers to evaluate postoperative patient reported outcomes following laparoscopic MALR. The questionnaire underwent initial assessment of content and face validity by regional experts (i.e., gastroenterologists and vascular surgeons), and survey items were updated based on this feedback. The survey was piloted on a patient with MALS who had undergone laparoscopic MALR outside of the study period.

### Outcomes

The primary outcome was change in subjective MALS symptoms from the preoperative period to the postoperative period as measured by the 14-question telephone survey. Secondary outcomes included operative time in minutes, postoperative length of stay in days, and 30-day postoperative morbidity. Patient characteristics including demographic variables, pre-existing comorbidities, pre-operative investigations, peri-operative outcomes (adverse events and length of stay), and postoperative outcomes (patient report symptoms during clinical follow-up, readmission rates, and emergency department visits) were recorded. Survey responses were recorded as a qualitative assessment of patient symptomatology and satisfaction.

### Statistical analysis

Data were collected via patient charts through an electronic medical record system established at St. Joseph's Healthcare. The patient population and outcomes where characterized using frequencies, means and standard deviations. Survey results were interpreted qualitatively. All collected data were recorded in a data collection sheet designed *a priori.* Stata statistical software (StataCorp, version 15; College Station, TX) was used to analyze iterative changes in operative time using a non-parametric Spearman rank correlation test for trend across ordered groups.

## Results

Overall, a total of nine patients underwent laparoscopic MALR at our institution during the study period. Patient characteristics are shown in Table 1, all procedures were performed laparoscopically with no conversions to open.

**Table 1:** Patient characteristics (SD, standard deviation; BMI, body mass index; GERD, gastroesophageal reflux disease; PUD, peptic ulcer disease; CTA, computed tomography angiography; ASA, American Society of Anesthesiologists; PTSD, post-traumatic stress disorder; MDD, major depressive disorder; BPD, borderline personality disorder; OCD, obsessive-compulsive disorder; ARFID, avoidant-restrictive food intake disorder).

	Number or Mean (+/- SD)
Total Completed Elective Procedures	9
Age at surgery (years)	37 +/- 15.63
Gender	
Male	0
Female	9
	
BMI	19.25 +/- 3.01
	
Comorbidities	
Chronic Pain	3
Previous eating disorder	2
Psychiatric disorder	6
PTSD	2
MDD	1
BPD	1
OCD	1
ARFID	1
GERD/PUD	6
Previous abdominal surgeries	4
	
Diagnostic Testing	
CTA	9
Vascular Ultrasound	1
Celiac Plexus block	7
	
ASA Score	
1	0
2	3
3	4
4	2

### Pre-operative characteristics

All patients in our study population were female with a mean age of 37.0 +/- 15.6 years. Chronic pain disorders were relevant in three of our patients, while six patients had co-existing psychiatric diagnoses. In addition, two patients had been previously diagnosed with eating disorders. Pre-operatively, two patients required supplemental nutrition, one via tube feeds and one requiring total parental nutrition. Five patients in our population had previous abdominal surgeries including cholecystectomy (n=1), appendectomy (n=1), caesarean section (n=1), hysterectomy (n=1), and one patient had a complicated surgical history with previous total colectomy and ileal pouch anal anastomosis for ulcerative colitis with a number of subsequent operations for pouch torsion and bowel obstructions resulting in an end ileostomy.

All of our patients underwent computed tomography angiography that demonstrated celiac artery compression, four patients also had findings suggestive of superior mesenteric artery syndrome. Seven patients underwent pre-operative celiac plexus block by interventional radiology or gastroenterology and experienced immediate relief of symptoms and were subsequently advanced to surgery.

Six of the included patients reported postprandial abdominal pain as their predominant preoperative symptom. Of the three remaining patients, two reported generalized abdominal pain and one reported nausea and vomiting as their predominant symptoms.

### Peri-operative characteristics

All patients underwent technically successful laparoscopic MALR with no conversions to open. There were no reported intra-operative complications. Mean surgical operative time was 92.4 +/- 31.4 minutes. Assessing operative times per case, revealed a statistically significant reduction in operative time as case numbers rose (p=0.046), operative times appeared to significantly decrease after the second operation (Table 2).

**Table 2:** Operating times per case (OR, operating room; mins, minutes; SD, standard deviation).

	**Case Number**										
	**1**	**2**	**3**	**4**	**5**	**6**	**7**	**8**	**9**	**Mean (SD)**	***P* value**
**OR Time [mins]**	133	157	78	85	81	70	66	91	71	92.4 (+/-31.37)	
											0.046*

Mean length of stay was 2.1 +/- 2.0 days. Five patients were discharged on postoperative day one. One patient had prolonged stay in hospital (seven days) secondary to urinary retention, and ongoing pain and nausea. One patient was cleared from surgical perspective on postoperative day two, however required repatriation to their home hospital for management of her home methadone dosing for chronic pain.

### Survey results

Patients were contacted via the previously described telephone survey to assess postoperative outcomes. The mean follow-up time was 71.4 +/- 36.5 weeks after surgery. Six out of nine patients described their pre-operative symptoms as “excruciating”. All patients experienced an improvement immediately postoperatively. Three patients reported complete resolution of symptoms, four patients reported partial improvement in symptoms, one patient reported moderate improvement, while one patient reported initial improvement followed by overall worsening of symptoms. Four patients endorsed ongoing improvement in symptoms, while five reported improvements over a period of weeks or months and then recurrence of symptoms thereafter. At the time of survey administration, five patients reported current pain scores on a scale of 1-10 as 6 or higher. Overall satisfaction with surgery was 68.9 +/- 29.3%, with seven patients stating they would still do surgery knowing what they know now. Seven patients reported improvement in their oral intake following surgery, whereas two reported worsening of their oral intake.

## Discussion

MALS is a rare clinical entity that causes compression of the celiac axis and remains both difficult to diagnose and manage. Historically, MALS was treated with decompression via laparotomy. However, this portends significant peri-operative morbidity. Recently, laparoscopy has emerged with a handful of case series and institutional experiences, reporting good outcomes however also a 12-30% conversion rate to open. In our study, we demonstrate successful technical releases via an entirely laparoscopic approach with no conversions to open in nine patients with MALS. Moreover, our series demonstrated a quick learning curve for an advanced laparoscopic surgeon, with surgical times significantly decreasing following the second case and an average surgical time of 92.4 +/- 31.4 minutes. Patient satisfaction in our study was moderate, with overall satisfaction of 68.9%. All patients experienced improvement in their symptoms immediately following surgery, however these improvements had varying degrees of duration.

The pathophysiology underlying MALS is still poorly understood and thought to be both ischemic and neurogenic in nature. However, a majority of patients appear to experience relief with MALR alone without celiac artery reconstruction suggesting this is primarily a neurogenic process. This is contrasted by a large series evaluating open surgical MALR, which included 35 patients, demonstrating that patients who underwent MALR and celiac artery revascularization had less late symptom recurrence than those undergoing MALR alone (24 vs 44%) [9]. However, in the same series, seven patients with severe recurrence underwent endovascular angioplasty and/or stenting, with only one patient reporting improvement in pain. Perhaps suggesting that the neurogenic nature of this disease process is at the very least the most dominant pathophysiological factor [13].

Late symptom recurrence appears to be similar between laparoscopic and open release, a recent series reported 36% of patients experiencing pain recurrence at a median of 6 months following either open, laparoscopic or robotic release with no difference attributable to surgical approach. The series by Reilly *et al.* identified certain factors with poor clinical improvement following surgery including: atypical pain, history of psychiatric disorders, age older than 60 and weight loss less than twenty pounds [9]. In our series, 5/9 patients reported recurrence of symptoms after initial improvement. Interestingly, the majority (6 out of 9) of patients had co-existing psychiatric disorders, thus perhaps influencing our observed satisfaction rates and self-reported improvement in symptoms following surgery. Further nuanced data pertaining to the timing of psychiatric diagnoses relative to the diagnosis and treatment of MALS is thus relevant, not only to determine their potential impact on quality of life data following MALR, but also to adequately manage these often concomitant disease processes.

Given that the pathophysiology and patterns of recurrence of MALS is not fully understood, in addition to the significant overlap between the symptoms present in MALS and other pathologies, a large focus has been on appropriate patient selection for surgical release as failures are possibly attributable to initial misdiagnoses. Our institutional approach involved a multidisciplinary team as well as pre-operative celiac axis block, to both aid in diagnosis and predict potential benefits following surgery. This has been recently supported by a single institution series demonstrating significant improvement in symptoms following celiac axis block, and no association with celiac artery abnormalities [14]. Additional studies are required to determine its true utility in predicting benefit from surgical release. Our institutions MALS assessment protocol is highlighted in Appendix 2.

The present study also highlights the rapid learning curve for skilled minimally invasive surgeons, with a significant reduction in operative time seen after just two cases. Moreover, four of the included patients (44.4%) had a history of prior intra-abdominal surgery. Thus, it is likely that laparoscopic MALR remains a safe approach in patients with preserved bursa omentalis. Repeat MALR for ongoing symptoms with radiographic evidence of ongoing celiac axis compression has yet to be studied extensively, with only a single patient in a large cohort published by Kazmi *et al.* having undergone repeat open MALR.[15] Further study evaluating the safety of repeat laparoscopic MALR would likely benefit the current body of literature.

The strengths of present series are that it details a robust assessment of patients pre-operatively prior to advancement to laparoscopy MALR and the use of patient reported outcomes following surgery. Our study has several limitations. It is a single arm study thereby not allowing for direct comparison between laparoscopic and open or between MALR with and without celiac artery revascularization. The small sample size limits our power in evaluating recurrence rates, rates of intraoperative complications, and rates of postoperative morbidity. Additionally, the patients included in the present study were the first to be enrolled in our institutions laparoscopic MALR program and preoperative pathway and thus deviations and changes to the protocol were likely to have occurred, increasing our within study heterogeneity. For example, the first two patients enrolled did not undergo celiac plexus blocks. Lastly, our patient survey is not standardized and captured heterogenous qualitative data that can be difficult to interpret with respect to surgical outcomes, as well as limits the generalizability of our findings.

## Conclusions

This case series highlights that laparoscopy has emerged as a safe and minimally invasive approach to MALR. However, longer term, properly powered data sets are required in order to further assess outcomes. Larger scale studies are required to confirm these findings and elucidate factors influencing post-operative outcomes.

## Source of funding

This research was conducted without external or internal sources of funding.

## Author's Contributions

Conception and design of the study – All authors

Acquisition of data – Mann, McKechnie

Analysis and interpretation of data – All authors

Drafting and revision of manuscript – All authors

Approval of the final version of the manuscript – All authors

## Declaration of Competing Interest

None declared.

## Appendix 1

**Telephone survey: Postop median arcuate ligament release outcomes**

1 On a scale of 0-10, how is your current pain or symptoms? 0 would be no pain or symptoms and 10 would be excruciating pain/ symptoms.
2 How do your symptoms compare to preoperatively? Preoperatively: 0-10 present symptoms: 0-10
  a What is you most bothersome symptom now?
  b What was your most bothersome symptom preoperatively?
3 To what degree did your symptoms improve following surgery:
  a No improvement
  b Partially better
  c Much better
  d Completely resolved
  e Worsened
4 If you experienced an improvement in symptoms, how long did the improvement last?
  a Weeks
  b Months
  c Ongoing
  d Still getting better
  e Got better for a while, now worsening
5 How satisfied are you with the surgery? 0-100%
6 Knowing now how you feel, would you do the surgery again? Y / N
  a What do you wish you had known preoperatively about the surgery that you would want others to know?
7 Have other medical conditions been diagnosed since your MALS surgery? What are they?
8 Have you had other procedures or surgeries completed since your MALS surgery?
9 Have you been able to decrease any medications because of having the MALS surgery?
10 Have you needed more medication since your MALS surgery?
11 Preoperatively could you eat a regular diet? Y / N
  a Did you need supplements like Boost/ Ensure?
  b Did you have a feeding tube?
  c Were you getting TPN?
12 After surgery has your diet changed? Better/ unchanged/ worse
13 In terms of day to day activities, how much can you do compared to before your surgery?
  a Less than before surgery
  b The same as before surgery
  c A bit more than before surgery
  d Much more than before surgery but not everything
  e I can do everything I would normally do
14 Were you working or in school before the MALS surgery?
  a Have you been able to return to work/ school since surgery?
  b If not, what has stopped you from being able to return to school/ work?

## Appendix 2

**MALS assessment protocol**

1. Multi-disciplinary assessment
  1.%2 Requires GI, Vascular General Surgical assessment for determining if this is a clinically relevant radiographic finding.
  2. The surgeons involved should have an appreciation of functional disorders and the likelihood of ongoing management by physicians and allied health providers even after the release.
  3. Preoperative assessment by a dietician is important and will likely be needed after surgery as well; if there are components of food avoidance, functional abdominal pain, then assessment by a psychologist, psychiatrist and eating disorders expert is involved for further assessment and help with management.
2 Physical exam
  1. Rule out abdominal wall pathology (Carnett's sign) and to assess for hyperalgesia.
3 DDx:
  1. Gastric pathology: gastritis; PUD, gastroparesis
  2. Duodenal: duodenitis, Celiac disease, duodenal ulcer disease, SMA syndrome
  3. Pancreatic disorders: pancreatitis
  4. Biliary: biliary colic, acute cholecystitis, biliary dyskinesia
  5. Functional disorders: functional dyspepsia, functional abdominal pain
4 EGD
5 CT angiogram
  1. Reviewed by Vascular Surgeon and Radiologist
6 Trial of Celiac plexus block
  1. Interventional radiology or interventional GI
7 If good response to the block, will discuss laparoscopic release.
  1. Outcomes show 50-60% improvement of some symptoms.
  2. Included in discussion of surgery and risks is what the patient could expect and what will be unlikely to improve. This is key, since a number of patients have overlapping symptoms, and most patients report some improvement in the short term. Long-term results are few.
